# Assessment of Peanut Protein Powder Quality by Near-Infrared Spectroscopy and Generalized Regression Neural Network-Based Approach

**DOI:** 10.3390/foods13111722

**Published:** 2024-05-31

**Authors:** Haofan Cui, Fengying Gu, Jingjing Qin, Zhenyuan Li, Yu Zhang, Qin Guo, Qiang Wang

**Affiliations:** 1 Institute of Food Science and Technology, Chinese Academy of Agricultural Sciences, Key Laboratory of Agro-Products Processing, Ministry of Agriculture, Beijing 100193, China; cuihaofan1999@163.com (H.C.); fengyinggfy@163.com (F.G.); qjj12250@163.com (J.Q.); li_zhenyuan@163.com (Z.L.); guoqin2010yl@163.com (Q.G.); 2 Biotechnology Research Institute, Chinese Academy of Agricultural Sciences, Ministry of Agriculture, Beijing 100081, China; zhangyu01@caas.cn

**Keywords:** peanut protein powder, near-infrared spectroscopy, partial least squares, generalized regression neural network, quality detection

## Abstract

The global demand for protein is on an upward trajectory, and peanut protein powder has emerged as a significant player, owing to its affordability and high quality, with great future market potential. However, the industry currently lacks efficient methods for rapid quality testing. This research paper addressed this gap by introducing a portable device with employed near-infrared spectroscopy (NIR) to quickly assess the quality of peanut protein powder. The principal component analysis (PCA), partial least squares (PLS), and generalized regression neural network (GRNN) methods were used to construct the model to further enhance the accuracy and efficiency of the device. The results demonstrated that the newly established NIR method with PLS and GRNN analysis simultaneously predicted the fat, protein, and moisture of peanut protein powder. The GRNN model showed better predictive performance than the PLS model, the correlation coefficient in calibration (Rcal) of the fat, the protein, and the moisture of peanut protein powder were 0.995, 0.990, and 0.990, respectively, and the residual prediction deviation (RPD) were 10.82, 10.03, and 8.41, respectively. The findings unveiled that the portable NIR spectroscopic equipment combined with the GRNN method achieved rapid quantitative analysis of peanut protein powder. This advancement holds a significant application of this device for the industry, potentially revolutionizing quality testing procedures and ensuring the consistent delivery of high-quality products to fulfil consumer desires.

## 1. Introduction

The global protein market is witnessing a significant surge in demand and is expected to reach $17.4 billion by the year 2027. This escalating demand is primarily driven by the recognition of the health benefits associated with protein consumption. Plant protein has gained considerable attention due to its affordability and diverse sources, which include beans, cereals, and oilseeds [1,2,3]. These proteins are not only cost-effective but also provide lipid-lowering properties and prevention of cardiovascular and cerebrovascular diseases [4,5,6]. For many years, soya has been the dominant player in the plant protein market. However, concerns regarding genetic modification have led to a shift in consumer focus towards other plant sources. Peanuts have emerged as a healthy and popular alternative source of plant protein [7,8,9]. In 2022, global peanut production reached an impressive 50.27 million tons, with 40% yield used for oil extraction. The production of peanut meal with both high and low temperature has reached to 7.98 million tons. High-temperature peanut meal is primarily used for feed, while low-temperature peanut meal is used to prepare peanut protein powder, which boasts a protein content of 50% [10]. Peanut protein powder is highly valued for its nutritional efficiency (58), ease of absorption by the human body (with a digestibility close to 90%), and unique peanut aroma [11]. It is widely used in a variety of food products, including beverages, meat products, and flour products [12,13]. Despite its popularity, the quality of peanut protein powder available in the market varies significantly. There is a pressing need for rapid and standardized quality evaluation methods to ensure consumers receive high-quality products.

The evaluation of protein powder quality employs a variety of methods, including chemical techniques such as the Kjeldahl method, Soxhlet extraction, and direct drying as well as near-infrared (NIR) spectroscopy and high-performance liquid chromatography. Among these, NIR spectroscopy has gained considerable interest from researchers due to its non-destructive nature and its ability to deliver fast and straightforward results [14,15,16,17]. The main indicators for evaluating the quality of protein powder include protein, fat, moisture, and trace substances like crude fiber, ash, vitamins, amino acids, trace elements, and carbohydrates [10,18]. Furthermore, both enterprises and consumers are mainly concerned about the protein content during the procurement and purchasing process. In addition, they pay significant attention to control the fat and moisture content during the production, storage, and sales process for the maintenance of the product’s quality and shelf life [19]. The Kjeldahl, Soxhlet extraction, and direct drying methods were utilized in a research study to evaluate the protein, fat, and moisture contents of peanut protein powder [20]. The results demonstrated several drawbacks, including being time-consuming, requiring complex sample pretreatment, and the use of organic reagents. Recently, near-infrared spectroscopy in conjunction with principal component regression (PCR), partial least squares regression (PLS), support vector machine regression (SVR) [21], and generalized regression neural network (GRNN) methods has been employed to monitor the quality of powdered samples, including insect powder [22], wheat powder [23], and cottonseed powder [24]. So far, the PLS methods have not been applied in the quality detection of peanut protein powder [25]. Therefore, there is an urgent need to establish a rapid evaluation method for assessing the quality of peanut protein powder. This would not only ensure the delivery of high-quality products to consumers but also streamline the production and sales process for enterprises. The development and implementation of such a method would contribute significantly to the protein powder industry.

In this study, a comprehensive evaluation method was used to assess the quality of peanut protein powder. A total of 51 peanut varieties were selected from China’s main planting areas, with 31 high-oleic peanut varieties included. The peanut protein powder was prepared using a low-temperature physical pressing process to retain the nutrients in the peanuts. Various processes were employed in the preparation of peanut protein powder during the sample selection stage, utilizing different production line models. To the best of our knowledge, it is the first instance in the literature of integrating peanut protein powder with multiple varieties, processes, and commercial products. This multifaceted approach was crucial in ensuring a thorough and accurate assessment of the product quality. The determination model for protein, fat, and water content in peanut protein powder was established by combining PLS and GRNN, presenting a novel method for evaluating the quality of peanut protein powder. Importantly, the PLS model and the GRNN model were applied for the first time in the detection of peanut protein powders, and the GRNN model showcased superior performance compared to the PLS model in predicting the primary components of peanut protein powder. Through the integration of these advanced analysis technologies, the accuracy and reliability of quality assessment have been significantly enhanced.

## 2. Materials and Methods

### 2.1. Materials

A collection of 51 peanut varieties, as shown in Table 1, encompassing both common peanuts (20 varieties) and high-oleic acid peanuts (31 varieties), were harvested over a span from 2019 to 2023. These samples were obtained from eight principal peanut-cultivating provinces across China (Henan, Hebei, Shandong, Liaoning, Fujian, Sichuan, Guangdong, and the Xinjiang Uygur Autonomous Region). This selection was carefully curated to ensure a broad representation of the varieties available. The samples were well preserved in a refrigerated environment maintained at 4 °C to safeguard their quality for subsequent utilization [26].

### 2.2. Peanut Protein Powder Collection

**Figure 1:**
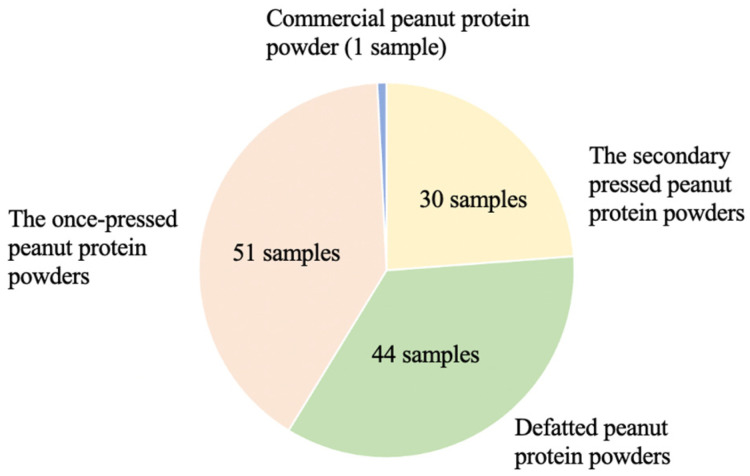
Distribution of peanut protein powder samples.

The process of preparing peanut protein powder from 51 varieties of peanut seeds was carried out. Each variety was subjected to weighing 500 g and underwent a thorough soaking in water for 30 min. Then, the red coats were delicately removed, and the seeds were allowed to dry. Peanut protein powder was prepared using an automatic hydraulic oil press (QY-230, Liangjunyiyou Machinery Co., Ltd., Qingdao, China). From initial pressing, 51 portions of once-pressed peanut meals were obtained. A subset of 30 peanut varieties were selected for secondary pressing, yielding 30 portions of the secondary-pressed peanut meals. These peanut meals were crushed using a crusher (FW80, Tester Instrument Co., Ltd., Tianjin, China) and sieved through a 40-mesh sieve to achieve uniformity in the sample state. Then, 44 peanut varieties were selected for defatting, resulting in the production of defatted peanut protein powders [27]. A total of 125 peanut protein powder samples were obtained, and each sample was stored at 4 °C in a refrigerator after being sealed in bags to preserve their integrity. Commercially available peanut protein powder (Jinsheng Cereals and Oils Group Co., Ltd., Linyi city, China) was added to the samples at the time of sample collection to make the peanut protein powder samples more representative (Figure 1). A total of 126 peanut protein powder samples were added to the commercially available peanut protein powder (Jinsheng Grain and Oil Group Co., Ltd., Linyi city, China) during sample collection. All samples were placed into the sealed bags and maintained at a refrigerated temperature to ensure their preservation.

### 2.3. Compositional Analysis

#### 2.3.1. Fat Determination

The quantification of fat content in the samples was conducted using the Soxhlet extraction method following the standard protocol (GB5009.6, 2016) [28]. The analytical procedure was performed with a Soxtec 2050 instrument (FOSS, Hillerød, Denmark).

#### 2.3.2. Protein Content Determination

The determination of protein content in the samples was carried out employing the Kjeldahl method, as specified in the standard protocol (GB5009.5, 2016) [29]. This method involved the use of a 2300 nitrogen analyzer with a conversion coefficient (6.25) (FOSS, Hillerød, Denmark). This coefficient assumes that the average nitrogen content of proteins is approximately 16%.

#### 2.3.3. Moisture Content Determination

The measurement of moisture content in the samples was conducted using the HE53 Halogen Moisture Analyzer (Mettler-Toledo, LLC. Columbus, OH, USA). The HE53 Halogen Moisture Analyzer is a valuable tool to effectively employ analytical techniques, offering quick and reliable results for moisture content analysis.

### 2.4. Near-Infrared Spectrum Acquisition

**Figure 2:**
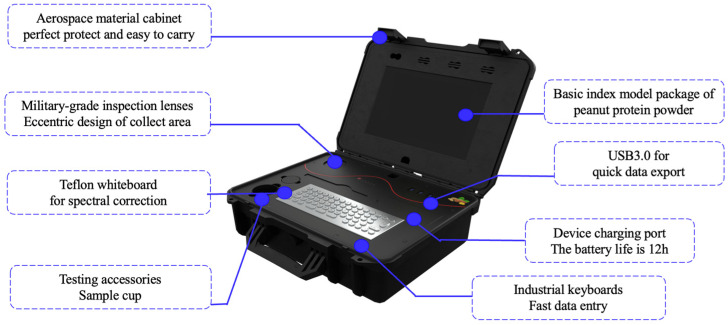
Near-infrared spectroscopy equipment used in this study.

The spectral data acquisition was performed by using a portable high-throughput peanut quality analyzer, a novel device developed by Wang Qiang’s team at the Chinese Academy of Agricultural Sciences (Figure 2) [30]. The innovative use of a portable near-infrared device marked a significant advancement in the scanning of powdered substances. This device comprised a Micro-NIRS 1700 spectrometer (VIAVI Solutions, San Jose, CA, USA). The portable tachymeter was subjected to a warm-up period of 30 min. This step was essential for the calibration of the dark current and the correction of the polytetrafluoroethylene (PTFE) whiteboard to eliminate the environmental interference that could potentially affect the detection results. The specifications for the portable high-throughput peanut quality analyzer are reported in Table 2. To ensure the integrity of the samples and the consistency of the testing environment, the peanut protein powder samples were carefully acclimatized from refrigeration to room temperature. Approximately 40–80 g of each peanut protein powder sample was placed in 3/4 of the sample cup (height 50 mm; diameter 51 mm). Great care was taken to ensure the cleanliness of the sample cup before experimentation. Afterward, each sample underwent three scans during the pouring phase, with the sample cup being rotated to guarantee that the samples were thoroughly scanned. The operation was repeated 3 times to obtain an average spectral reading. The peanuts protein powder was refilled into the sample cup during the second and third scans to obtain multiple near-infrared scanning spectral information of the same sample. The spectral data was collected using the Micro-NIR Pro 2.4 software (VIAVI Solutions, USA) [31].

### 2.5. Principal Component Analysis

Principal component analysis is a statistical technique, widely used for the feature extraction of spectral data. PCA transforms the original set of variables into a new coordinate system. The first large variance of any projection of the data comes on the first coordinate, which is the first principal component, and the process continues for subsequent principal components. To establish the data set, PCA could identify both outliers and multiple indicators that were converted into a more manageable number of principal components. The PCA step was executed using the Matlab software (Matlab R2021b, Mathworks Inc., Natick, MA, USA) [32].

### 2.6. Spectral Preprocessing

Spectral preprocessing was performed to minimize the impact of the sample set on the NIR spectra to the greatest extent possible. It reduced the interference factors, such as natural light, which in turn enhanced the stability of the model and the accuracy of the data. The spectral curves of the sample set may exhibit duplication, baseline drift, dispersion, and other phenomena, such as uneven sample distribution or external environmental influences. To address these issues, a suite of eight preprocessing methods was employed to eliminate errors in the original spectral data. These methods included the normalization (normalize), first-order derivative (first derivative, FD), second-order derivative processing (second derivative, SD), the baseline calibration (baseline), detrend, multiplicative scatter correction (MSC), and deresolve [33,34]. The reasonable preprocessing of NIR spectra effectively filtered the noise information in NIR spectra, reduced the quantitative analysis complexity of NIR models, and improved the stability of the models. The detrend method was particularly used to eliminate the baseline drift in the original spectra. Both MSC and SNV served similar purposes, which were used to eliminate the scattering caused by the distribution and size of the particles within the samples [35]. Derivative spectra processing eliminated the baseline and background interferences effectively. The outliers were eliminated by PCA. This comprehensive approach to preprocessing ensures that the NIR spectroscopy models are robust and reliable, as highlighted in the studies of Zhao et al. [33] and Mishra [35].

### 2.7. Model Establishment

The spectral data of the peanut samples were expressed as independent variables, while the chemical indicators of each sample were recorded as dependent variables. To analyze the data, two advanced statistical modeling techniques were used: partial least squares regression (PLS) and the generalized neural network model (GRNN). The Unscrambler X 10.4 (CAMO Software AS, Oslo, Norway) was utilized to establish the PLS models. MATLAB (version R2021b, Math Works Inc., Natick, MA, USA) was utilized to establish the GRNN models.

PLS is a regression modeling method, which considers dependent variables in relation to independent variables. The algorithm aimed to extract as many principal components as possible from independent variables and dependent variables while maximizing the correlation between these components. PLS facilitated the construction of highly accurate models, which combined the advantages of the three analyses, such as typical correlation analysis, canonical correlation analysis, and multiple linear regression analysis [36,37].

The generalized regression neural network (GRNN) is a kind of radial basis function neural network known for its potent non-linear mapping capabilities and rapid learning speed. GRNN is particularly effective when dealing with small-sample datasets, which includes a strong non-linear mapping ability and learning speed. The GRNN model comprises four layers: the input layer, pattern layer, summation layer, and output layer. The GRNN can minimize the influence of artificial subjective assumptions on the prediction outcomes. The network’s simple structure allows for fast computation speeds and is not affected by the multiple covariance of input data.

### 2.8. Method of Evaluating Model

The efficiency of the model is a key aspect of the entire study, representing the performance of the model. The model’s effectiveness is typically assessed using various indicators, such as the correlation coefficient in calibration (Rcal), standard error in calibration (SEC), correlation coefficient in validation (Rcv), standard error in validation (SECV), correlation coefficient in prediction (Rcp), standard error in prediction (SEP), and residual prediction deviation (RPD), which determines the accuracy of the model’s predictions during calibration, as outlined in the research conducted by Lima et al. [38]. The best model is characterized by higher Rcal/Rcv values (with 1 being the ideal) and lower SEC/SEP values. Along with R values, other statistical metrics like residual prediction deviation (RPD) play a vital role in model assessment. RPD is determined by dividing the standard deviation (SD) of the prediction set by SEP. An RPD below 1.5 is considered unsuitable, while models with an RPD exceeding 2 are deemed outstanding [39,40,41]. The careful consideration of these evaluation metrics is essential for the validation and refinement of the predictive models in the study.

## 3. Results

### 3.1. Chemical Indexes Analysis of Peanut Protein Powder

**Figure 3:**
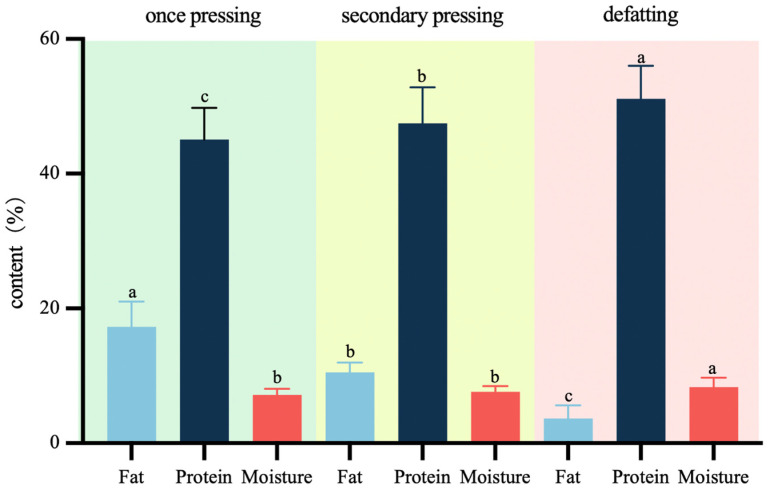
The fat, protein, and moisture content of peanut protein powder under different treatments. (Different letters in the same column indicated significant differences at *p* < 0.05).

Currently, the market offers peanut protein powder with a wide range of fat content, which is greatly influenced by different production processes, such as pressing times, type of oil press used, and the degreasing process. The application of degreasing treatment to peanut protein powder can effectively promote its shelf life. The protein content of peanut protein powder available on the market typically ranges from 35% to 60%, with a moisture content of less than or equal to 10% [10]. To ensure the model’s adaptability to diverse production environments and processes, this study employed a three-step pressing process. The one-time pressing resulted in various indicators for peanut protein powders, including residual oil content ranging from 12.97% to 29.17% and protein content ranging from 36.19% to 52.28%. Secondary-pressed peanut protein powders exhibited residual oil content between 7.95% and 12.81%, as well as protein content ranging from 39.00% to 58.20%. Defatted peanut protein powders showed residual oil content ranging from 0.83% to 7.74% and protein content ranging from 42.00% to 58.78%. This approach facilitated a broader distribution of fat and protein content, enhancing the model’s predictive capabilities. As shown in Figure 3, the fat content of peanut protein powder was significantly reduced following the secondary pressing and degreasing processes, while the protein content was increased. The moisture content appeared to be relatively unaffected by the pressing conditions.

In this study, 126 samples of peanut protein powder were prepared, and the average values of fat, protein, and moisture contents of the samples are reported in Table 3. The fat content varied from 0.83% to 29.17%, the protein content ranged from 36.19% to 58.78%, and the moisture content ranged between 5.34% to 11.60%. The fat and protein content of the samples were able to completely cover the commercially available protein powders, including with high, low, and medium contents. The samples with a moisture content greater than 10% were also included in the training set and proved beneficial for monitoring the production and processing of peanut protein powder. Moisture content was an important index of peanut quality and storage stability. It was an important parameter that should be measured, monitored, and controlled during harvesting, drying, processing, marketing, and storage. It enabled the timely detection and handling of abnormal water samples.

The samples were found to be well-representative. A study by Liu et al. [11] found that the protein content of peanut meal, a by-product of oil extraction, is about 55%, the range of peanut protein powder content in its determination. The protein, lipid, and water contents of the peanut protein powder determined in another study by Zhang et al. [42] were 55.3% (dry basis), 8.8% (dry basis), and 6.1%, respectively. These studies provided valuable insights into the composition of peanut protein powder. The quality indicators of peanut protein powder depended on the specific conditions and parameters used in the production process. The quality index of peanut protein powder in this study had a wide range, which can encompass most of the quality of peanut protein powder.

### 3.2. Near-Infrared (NIR) Spectral Data

**Figure 4:**
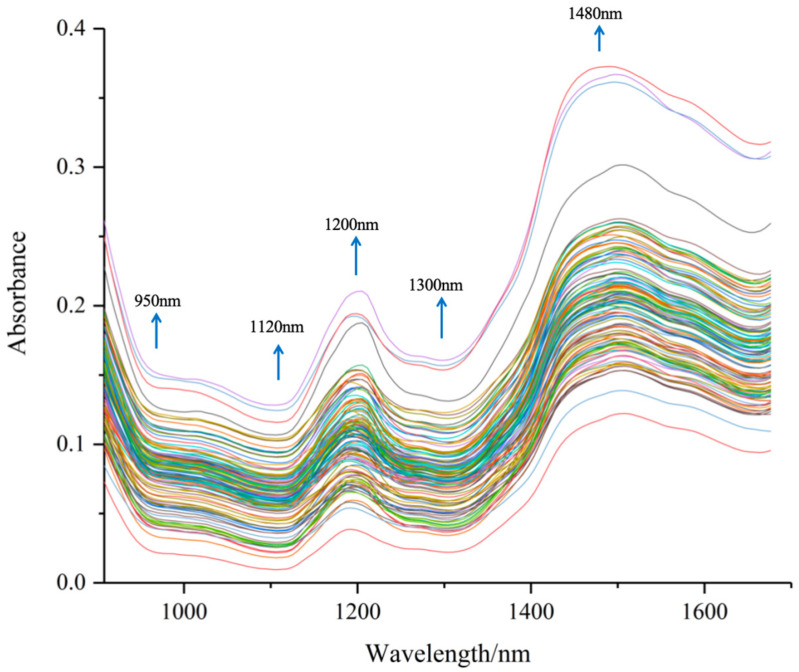
Original spectra of peanut protein powders.

The spectral data of 126 peanut protein powder samples are shown in Figure 4. The figure shows the spectrum of different peanut varieties under different preparation processes, the near-infrared spectra of various peanut protein powder samples show similar trends, but the absorption peak intensities of different samples are different, indicating that the components of different samples have differences, which can be used for the construction of the quantitative model of near-infrared spectroscopy.

The spectral curve of peanut protein powders was similar to the trend of the spectral curve of other cereal grain powder samples [43]. The original spectra exhibited peaks at 1200 and 1480 nm, while valleys were observed at 950, 1120, and 1300 nm The bands observed in the near-infrared region indicated the absorption of the hydrogen-containing groups (C–H, N–H, and O–H), from major components found in the peanut protein powders, such as water, lipids, and proteins.

From Figure 4, we can highlight the following spectral bands [26]:

950 nm: N–H stretch second overtone, proteins; O–H stretch second overtone, water;

1120 nm: C–H stretch second overtone, lipids;

1200 nm: O–H stretch + O–H deformation, water;

1300 nm: C–H combination: lipids;

1480 nm: N–H stretch first overtone, proteins; O–H stretch first overtone, water; C–H combination, lipids.

These groups provided rich structural and compositional information that reflected the unique characteristics of peanut protein powder. These characteristics were advantageous for subsequent model prediction. The spectral data provided a comprehensive overview of the inherent properties of peanut protein powder, offering valuable insights into its composition and structure. This information was instrumental in enhancing the predictive capabilities of the model, thereby facilitating a more accurate analysis of the peanut protein powder.

Near-infrared spectroscopy delivered invaluable data on the anharmonic nature of molecular vibrations and peculiarities of intermolecular interactions [44]. The spectral range chosen to develop a near-infrared spectroscopy model in this study closely resembled that used in detection models for substances like nuts and oilseeds. For instance, Zhao et al. [45] utilized hyperspectral images ranging from 950 to 1700 nm to establish a PLS model for the rapid quality control of peanut and walnut powder in flour. Similarly, Mohammad Akbar Faqeerzada et al. [46] employed a line scan hyperspectral imaging system covering a spectral range of 900 to 2494 nm to swiftly and non-destructively screen almond powder samples.

### 3.3. Principal Component Analysis (PCA)

**Figure 5:**
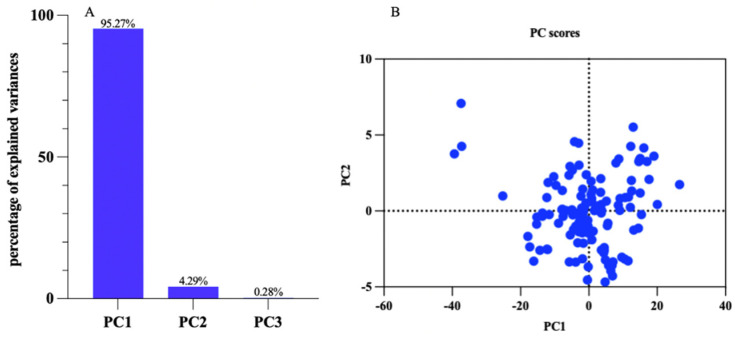
Principal component analysis diagram of the peanut protein powder samples. (**A**) Percentage of explained variances; (**B**) PC scores.

The essence of principal component analysis was feature extraction, which mainly reserved the main classification information of the original space to the maximum extent in the feature space. The dimension of the feature space was far lower than that of the original space. Without reducing the “effective” information, the original dataset was converted to “effective” information with fewer dimensions. Principal component analysis (PCA) was employed to analyze the wavelength values of 126 peanut protein powder samples. The principal component variance contribution rates of PC1, PC2, and PC3 were 95.27%, 4.29%, and 0.28%, respectively, and the cumulative contribution rate was 99.85%, which could cover the sample information, as shown in Figure 5. This high cumulative contribution rate showed that the three principal components (PC1, PC2, and PC3) could effectively cover the information of the sample data. In other words, these three components collected nearly all the variability in the data, thereby providing a comprehensive representation of the sample information.

PCA was usually used for feature information extraction and feature wavelength extraction, thereby improving the effectiveness of the training samples and improving recognition accuracy. A method for identifying the adulterated cocoa powder was presented by Yang et al. [47], where the relative areas of 12 common characteristic peaks in the fingerprints were processed using PCA. Zhang et al. [48] used PCA to study 33 representative traits associated with flavor and found that total sugar, sucrose, and total tocopherol had the most abundant information related to peanut flavor.

The PCA step can reduce the data matrix dimension and compress the data points into interpretable variables. Bilal et al. [49] used principal component scores as input variables and applied PCA and linear discriminant analysis (LDA) models to quantify peanuts.

### 3.4. PLS Model

**Figure 6:**
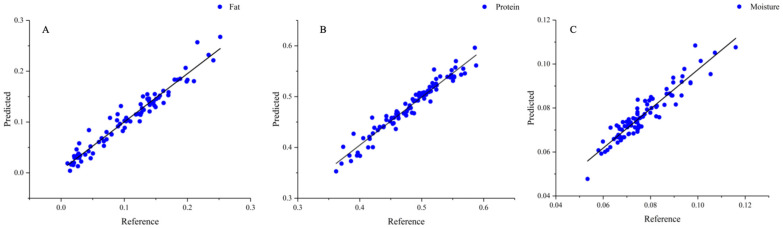
PLS model of peanut protein powder. (**A**) Fat; (**B**) protein; (**C**) moisture.

The chemical values of fat, protein, and moisture content of peanut protein powder were correlated with the near-infrared spectrum values. This was achieved using the Unscrambler X 10.4 software, which facilitated the screening of the optimal spectral preprocessing method, the selection of best principal components, and the construction of a model using the PLS. In this study, all data were divided in a 3:1 ratio and partitioned into calibration (95 samples) and prediction (31 samples). The best model was identified based on the correlation coefficient in prediction (Rcp) and standard error of prediction (SEP), with a high correlation coefficient and low error indicating high accuracy and stability. As shown in Table 4, it was observed that not all spectra improved the model performance after preprocessing. As shown in Figure 6, the optimal spectral pretreatment methods for PLS models of fat, protein, and moisture content of peanut protein powder were FD, SD, and SD, respectively. The model repeated cross-validation to eliminate outliers. The improvement was attributed to the enhancement of the absorption peaks of the spectra following the derivative processing and the reduction of the baseline offset. These adjustments made the model more sensitive to changes in the chemical composition and enhanced the performance of the model. The PLS method had been widely used in the analysis of peanuts and peanut products. Partial least squares discriminant analysis (PLS-DA) was employed by Song et al. [50] to create models that could distinguish between uncontaminated and aflatoxin-contaminated peanut oil. Another study [51] focused on the nutritional significance of peanuts, specifically examining the free amino acid (FAA) and crude protein (CP) content in raw peanut seeds.

The models were established to predict the fat, protein, and moisture content of peanut protein powder. As can be seen in Table 5, the model for fat content achieved a calibration correlation coefficient (Rcal) of 0.9750, and a standard error prediction (SEP) of 0.0129. The protein model had an Rcal of 0.9771 and an SEP of 0.0148, while the moisture model had an Rcal of 0.9428 and an SEP of 0.0038. The accuracy of these models was evaluated using the residual prediction deviation (RPD). An RPD greater than 1.4 indicated that the model can provide reasonable prediction results, while an RPD greater than 2 suggested that the model had a good prediction effect. The RPD of the three PLS models of peanut protein powder was greater than 2, proving that the constructed model had a high degree of reliability. Compared to the chemical method, the error in the three indicators was smaller, the prediction speed was faster, and the model was more robust, eliminating the possibility of random errors caused by large data fluctuations. A near-infrared protein powder detection model was established by Ingle et al. [52], which exhibited a correlation coefficient of 0.986. It used a training set of samples with a protein content of 20% to 90%, consisting of only 17 samples, and validated only 85% to 88% protein content in the protein powder. This was significantly much smaller than the more than 100 samples in this study, and the error reached 2%. In contrast, the error of the protein detection model in this study was reduced by 26%. These findings unveiled the potential of using near-infrared spectroscopy in combination with chemometric algorithms for the rapid and accurate prediction of the chemical composition of peanut protein powder.

### 3.5. Generalized Regression Neural Network (GRNN) Model

The chemical values of fat, protein, and moisture content in peanut protein powder were correlated with the near-infrared spectrum values. The model was established using the MATLAB R2021b software. The spectrum of peanut protein powder consisted of 125 wavelengths. However, the data information and sensitivity of the near-infrared spectrometer were affected by the operating environment of the equipment and the differences between different operators. Some redundant information was present in the spectral information, which led to low computational accuracy and poor model stability. Therefore, feature extraction of the original near-infrared spectra was extremely important, both to reduce the time of model calculation and to improve the stability of the model.

Prior to the neural network analysis of the data, the original data were extracted, and the first three principal components were extracted. Therefore, the cumulative variance contribution rate of the first three principal components was 99.85%, which can reflect the spectral information of peanut protein powders, representing that these three principal components reflected 99.85% of the spectral information contained in the original spectrum. For the 126 samples of peanut protein powder, 26 samples were first randomly selected as the test set and the remaining 100 samples as the training set. The principal factor scores after feature selection were used as the input variables of the model. This approach significantly reduced the time of training and the size of the network. In the GRNN model, the smoothing factor determined both the error of the training set and the shape of the hidden layer basis function, which directly affected the accuracy of the model prediction. The smoothing factor was determined by repeatedly comparing the selection of the smoothing factor with the output results. The fat (%), protein (%), and moisture content (%) of peanut protein powder were used as the outputs of the network, respectively, to construct the GRNN model. The optimal smoothing parameters were found to be 0.02, 0.015, and 0.02, respectively.

**Figure 7:**
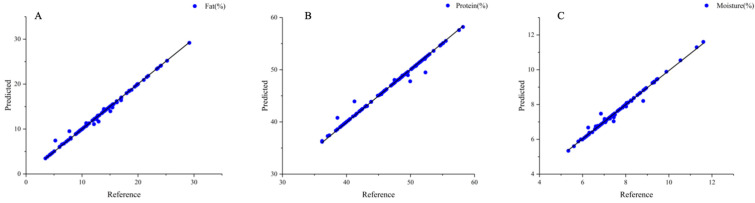
GRNN model of peanut protein powder. (**A**) Fat; (**B**) protein; (**C**) moisture.

The GRNN model of fat, protein, and moisture content exhibited better prediction results for their chemical values, as represented in Figure 7. The correlation coefficients of the training set were 0.9952, 0.9904, and 0.9896 for fat, protein, and moisture, respectively. The corresponding errors were 0.0022%, 0.0219%, and 0.0221%, and the RPD values were 10.82, 10.03, and 8.41, respectively (Table 6). When the RPD was greater than 10, the model was applied to real-time process control and optimization. The prediction results of the fat, protein, and moisture models established by GRNN were better than those of the PLS model. Compared with the PLS model, the GRNN model exhibited higher model accuracy, with improved correlation coefficients of the fat, protein, and moisture models, and substantially reduced errors. The correlation coefficients of the prediction reached more than 0.98, and the fat model had the lowest error of 0.0022%, which may be the result of the lower fat content of the samples in the validation set. Compared with the chemical measurement method, both PLS and the GRNN models demonstrated smaller errors for the three indicators, demonstrating more robust models, and less large data fluctuation due to random error. The errors in the GRNN detection model for soybean meal powder were 0.3% for protein and 0.2% for moisture content [53]. It was observed that the GRNN model had a good foundation in predicting the content of each component of the powder, and the GRNN method was applied to the quality detection of peanut protein powder. This study demonstrated the potential of using near-infrared spectroscopy in combination with neural network models for the rapid and accurate prediction of the chemical composition of peanut protein powder.

## 4. Conclusions

The integration of near-infrared spectroscopy with PLS and GRNN presented a rapid and efficient method for detecting the fat, protein, and moisture content of peanut protein powder. This approach facilitated the real-time process monitoring of peanut protein powder and provided a fast detection method for peanut food that ensured good peanut protein powder raw materials for the food industry. The implementation of near-infrared spectroscopy enhanced the speed and accuracy of detection and contributed to the optimization of the production process. In the future, the application of a near-infrared detection system can be extended to the peanut protein powder production line and processing system. This would enable continuous monitoring and quality control during the production process, thereby contributing significantly to the advancement of the peanut industry. By ensuring the consistent quality of peanut protein powder, this method can help manufacturers meet food safety standards and consumer expectations. Furthermore, the real-time data provided by this system can inform decision-making processes, enabling timely adjustments to the production process as needed. It can improve efficiency, reduce waste, and enhance product quality, all of which are critical for the sustainable growth of the peanut industry.

The direction of future development can be carried out from the following aspects: Firstly, the sample size can be expanded to include almond powder, soybean protein powder, and other common protein powders used in daily production and life. This expansion will enable the exploration of differences among various protein powders, leading to the establishment of a more universal protein powder-monitoring model. Such a model can then be applied to a wider array of research topics. Secondly, it is essential to delve into more detection indicators beyond the traditional evaluation based on fat, protein, and moisture content. In recent years, assessing ash content, digestibility, solubility, and other related indicators has gained increasing importance. By incorporating these additional indicators into the analysis, researchers can obtain a comprehensive understanding of protein powder quality. This multifaceted approach will not only enhance the depth but also broaden the scope of research results.

## Figures and Tables

**Table 1:** Peanut varieties and number.

No.	Variety Name	No.	Variety Name	No.	Variety Name
1	Jihua 1353	18	Jihua 13	35	Minhua 825
2	Jihua 443	19	Jihua 16	36	Puhua 28
3	Dawuxiaoguo	20	Jihua 18	37	Jihua 97
4	Fuhua 22	21	Jihua 19	38	Yuhua 93
5	Fuhua 24	22	Jihua 52	39	Yueyou 1826
6	Fuhua 35	23	Jihua 915	40	Qinghua 6
7	Heihuasheng	24	Weihua 30	41	Qinghua 308
8	Huayu 16	25	Puhua 85	42	Quanhonghua 1
9	Huayu 22	26	Huayu 666	43	Quanhua 551
10	Huayu 23	27	Jinonghua 20	44	Shanhua 13
11	Xvhuatian 29	28	Jinonghua 6	45	Silihong
12	Yuhua 22	29	Jihuatian 1	46	Tianfu 3
13	Yuhua 37	30	Kainong 1715	47	Tianfu 39
14	Huayu 917	31	Kainong 1760	48	Weihua 25
15	Huayu 9118	32	Kainong 308	49	Weihua 29
16	Qihua 1	33	Kainong 311	50	Yunhuasheng 15
17	Jihua 9	34	Kainong 61	51	Yuhua 65

The name of the peanut variety comes from the Chinese seed industry data platform. http://202.127.42.47:6010/index.aspx, accessed on 1 February 2024.

**Table 2:** Specifications for the portable high-throughput peanut quality analyzer.

Index	Details
Light source	Dual integrated vacuum tungsten lamps
Spectroscopic element	Linear Various Fitter (LVF)
Detector	InGaAs diode array
Wavelength range	900–1700 nm
Spectral bandwidth	Less than 1.25% of center wavelength
Display controller	Surface line of Microsoft Corp., Redmond, WA, USA.

**Table 3:** Chemical value analysis of peanut protein powders.

Component	Min	Max	Mean	S.D.	C.V.	P25	P50	P75
fat	0.83%	29.17%	10.81%	6.48%	59.92%	4.67%	10.8%	14.9%
protein	36.19%	58.78%	47.80%	5.56%	11.63%	43.8%	48.5%	51.8%
moisture	5.34%	11.60%	7.70%	1.17%	15.24%	6.89%	7.45%	8.34%

Coefficient of variation (C.V.), median (P50), 25th percentile (P25), and 75th percentile (P75).

**Table 4:** PLS model of peanut protein powder under different spectral pretreatments.

Pretreatment	Fat				Protein				Moisture			
	Rcal	SEC	Rcv	SECV	Rcal	SEC	Rcv	SECV	Rcal	SEC	Rcv	SECV
Raw spectrum	0.9041	0.0261	0.8924	0.0276	0.9393	0.0191	0.9249	0.0212	0.8973	0.0054	0.8618	0.0063
Normalize	0.9029	0.0263	0.8899	0.0279	0.9445	0.0183	0.9298	0.0206	0.8872	0.0057	0.8536	0.0064
FD	0.9080	0.0256	0.8896	0. 0280	0.9491	0.0176	0.9292	0.0207	0.9132	0.0050	0.8793	0.0059
SD	0.9064	0.0258	0.8896	0.0280	0.9542	0.0167	0.9331	0.0201	0.9169	0.0049	0.8746	0.0060
Baseline	0.9036	0.0262	0.8851	0.0285	0.9426	0.0186	0.9298	0.0206	0.9007	0.0053	0.8694	0.0061
SNV	0.9057	0.0260	0.8905	0.0279	0.9500	0.0174	0.9367	0.0196	0.9099	0.0051	0.8831	0.0058
Detrending	0.9021	0.0264	0.8840	0.0286	0.9429	0.0186	0.9294	0.0206	0.8980	0.0054	0.8607	0.0063
MSC	0.9056	0.0260	0.8904	0.0279	0.9499	0.0174	0.9368	0.0195	0.9092	0.0051	0.8827	0.0058
Deresolve	0.9037	0.0262	0.8919	0.0277	0.9383	0.0193	0.9238	0.0214	0.8964	0.0055	0.8613	0.0063

**Table 5:** Construction and validation of PLS model.

Component	Pretreatment	Factor	Calibration Set Sample	Rcal	SEC	Rcv	SECV	Validation Set Sample	Rcp	SEP	RPD
fat	FD	3	88	0.9750	0.0138	0.9695	0.0153	31	0.9849	0.0129	5.77
protein	SD	6	89	0.9771	0.0114	0.9571	0.0148	31	0.9660	0.0148	3.80
moisture	SD	6	89	0.9428	0.0039	0.9138	0.0048	26	0.9106	0.0038	2.37

**Table 6:** Construction and validation of the GRNN model.

Component	Smoothing Parameters	Rcal	SEC	Rcp	SEP	RPD
fat	0.02	0.9952	0.0145	0.9915	0.0022	10.82
protein	0.015	0.9904	0.0231	0.9900	0.0219	10.03
moisture	0.02	0.9896	0.0179	0.9859	0.0221	8.41

## Data Availability

The original contributions presented in the study are included in the article, further inquiries can be directed to the corresponding author.
